# Therapeutic Effects Comparison and Revision Case Analysis of Unicompartmental Knee Arthroplasty and Open Wedge High Tibial Osteotomy in Treating Medial Knee Osteoarthritis in Patients Under 60 years: A 2–6‐year Follow‐up Study

**DOI:** 10.1111/os.12761

**Published:** 2020-09-06

**Authors:** Zhang Ziqi, Mei Yufeng, Zhang Lei, Wang Chunsheng, Yang Pei, Wang Kunzheng

**Affiliations:** ^1^ Department of Bone and Joint Surgery The Second Affiliated Hospital of Xi'an Jiaotong University, Xi'an Jiaotong University Xi'an China; ^2^ Department of Rheumatology and Immune Joint Surgery Honghui Hospital, Xi'an Jiaotong University Xi'an China

**Keywords:** Medial knee osteoarthritis, Open wedge high tibial osteotomy, Outcome, Unicompartmental Knee arthroplasty

## Abstract

**Objective:**

To evaluate the therapeutic effects and revision cases of unicompartmental knee arthroplasty (UKA) and open wedge high tibial osteotomy (OWHTO) in treating medial knee osteoarthritis (MKOA) in patients under 60 years.

**Methods:**

The present retrospective study included a total of 192 patients who were diagnosed with MKOA and treated by UKA or OWHTO in the Second Affiliated hospital of Xi'an Jiaotong University and Xi'an Honghui Hospital between December 2012 and December 2016. Among these patients, 83 were treated by UKA (17 men and 66 women, aged 53.7 ± 5.2 years) and 109 were treated by OWHTO (23 men and 86 women, aged 51.8 ± 6.9 years). Patients were followed up at 1, 3, 6, and 12 months for the first year postoperation, and every 6 months from the second year postoperation. Basic data, perioperative data, hospital for special surgery (HSS) score, visual analogue pain score (VAS), low‐impact recovery, and revision cases of the patients were evaluated.

**Results:**

The average follow‐up periods of the UKA group and the OWHTO group were 39.3 ± 11.2 months and 40.2 ± 13.5 months, respectively. No significant difference was found in the basic data of the two groups (*P* ≥ 0.05). The operative time, incision length, and dominant blood loss of the UKA group was less than those of OWHTO group by 19.6%, 10.7%, and 35.1%, respectively, and the differences were significant (*P* < 0.05), while no significant difference was found in postoperative in‐bed time (*P* ≥ 0.05). The HSS scores of the UKA group at 1 and 3 months postoperation were higher than those of the OWHTO group by 5.1% and 3.9% (*P* < 0.05), while no differences were found from 6 months postoperation (*P* ≥ 0.05). The VAS score of the UKA group 1 month postoperation was lower than that of the OWHTO group by 12.2% (*P* < 0.05), while no differences were found from 3 months postoperation (*P* ≥ 0.05). One year after the operation, most patients in both groups could not achieve ideal recovery in low‐impact sports, and no significant differences were found (*P* < 0.05). The sport in which most patients could not achieve ideal recovery was mountain climbing. No revision cases occurred in the OWHTO group, while two revisions occurred in the UKA group.

**Conclusion:**

Candidates for UKA should be chosen carefully and the current indications and contraindications raised by Goodfellow should be modified.

## Introduction

Medial knee osteoarthritis (MKOA), which is generally characterized by degeneration of medial knee cartilage and the subsequent bone‐to‐bone wearing in medial knee unicompartment,^1^ is a common disease in the clinical work of joint surgery^2^. Pain on the medial unicompartmental knee, dysfunction, and malformation of the knee joint are the cardinal symptoms. For mild MKOA (Alhback stage I–II), conservative treatment, including oral medicines and physical therapy, can obtain ideal effects^3^, ^4^. However, for moderate and severe MKOA (Alhback stage III–IV), surgical operations are often necessary^5^, ^6^.

At present, the most frequently‐used operations for MKOA are unicompartmental knee arthroplasty (UKA) and open wedge high tibial osteotomy (OWHTO). It is reported that both UKA and OWHTO can obtain ideal outcomes for MKOA, including pain relief and function reconstruction^7^, ^8^, ^9^, ^10^.

However, there is still controversy regarding UKA and OWHTO both in regard to outcomes and operation options. Some researchers believe that OWHTO provides better physical activity outcomes for younger patients, whereas UKA is more suitable for older patients due to shorter rehabilitation time and faster functional recovery, while some researchers have found that UKA has a higher revision rate than OWHTO, which should be considered carefully before carrying out either procedure^11^, ^12^. In addition, some reports demonstrate that UKA results in significantly better functional outcomes and postoperative velocity, along with less postoperative pain and fewer postoperative complications^13^, ^14^. However, the outcomes of UKA vary in different reports. Crawford's report found that activity level does not affect survivorship of unicondylar knee arthroplasty,^15^ while Sever's report showed that UKA had higher early‐stage aseptic revision rates than TKA^16^. Furthermore, in Schroer's research, UKA is deemed a complete failure due to the unacceptably high revision rate, which led the author to refuse to use it anymore^17^.

In general, the controversies in previous reports on UKA and OWHTO are mainly focused on the following three points. First, does UKA or OWHTO have a better outcome for patients with MKOA? Second, besides age, what could be the differences between UKA and OWHTO in terms of candidate selection? Third, why do UKA outcomes in different reports vary? We designed the present study to solve these problems. We collected data on patients undergoing UKA and OWHTO in our department and Xi'an Honghui Hospital, evaluated the outcomes, and analyzed the revision cases.

The purposes of the present study are: (i) to compare the outcomes of UKA and OWHTO in treating MKOA; (ii) to reveal the critical factors in the choice of operation besides age; and (iii) to investigate why outcomes of UKA vary in different reports.

## Methods

### *Basic Data*

The study protocol was approved by the local institutional review board of the Second affiliated hospital of Xi'an Jiaotong University and Xi'an Honghui Hospital. Data on MKOA patients undergoing UKA or OWHTO in the two hospitals between December 2012 and December 2016 was collected. All patients were followed up at 1, 3, 6, and 12 months for the first year after the operations, and every 6 months from the second year. The inclusion and exclusion criteria are as follows.

Inclusion criteria were patients who were: (i) diagnosed with MKOA; and (ii) treated by UKA or OWHTO in our department or Xi'an Honghui Hospital between December 2012 and December 2016.

Exclusion criteria were: (i) patients undergoing one‐stage bilateral operations, such as bilateral UKA, bilateral OWHTO, and unilateral UKA with contralateral OWHTO; (ii) patients with body mass index (BMI) ≥ 35; (iii) age ≥ 60 years; (iv) patients who had anemia, hypertension, diabetes, myocardial infarction, cerebral infarction, coagulation disorders, or deep venous thrombosis (DVT) in the past 6 months; (v) patients with diseases that may affect knee function except MKOA, such as poliomyelitis, myasthenia, and nervous system disease; (vi) any lost followed‐up cases; and (vii) any cases whose operations were not carried out by chief surgeons.

There were 192 cases included in the present study in total: 83 cases of UKA (UKA group) and 109 cases of OWHTO (OWHTO group). There were 17 men and 66 women in the UKA group; the average age was 53.7 ± 5.2 years, the average BMI was 27.7 ± 4.1 Kg/m^2^, and the average follow up was 39.3 ± 11.2 months. There are 23 men and 86 women in the OWHTO group; the average age was 51.8 ± 6.9 years, the average BMI was 26.4 ± 3.6 Kg/m^2^, and the average follow up was 40.2 ± 13.5 months. No differences were found between the two groups in age, BMI, gender distribution, or follow‐up (*P* > 0.05). The basic data of the two groups are shown in Table 1.

**TABLE 1:** Comparison on the basic data between the two groups

	UKA group (*n* = 83)	OWHTO group (*n* = 109)	*P‐*value
Age (years)	53.7 ± 5.2	51.8 ± 6.9	0.308
BMI (Kg/m^2^)	27.7 ± 4.1	26.4 ± 3.6	0.207
Gender (M:F)	17:66	23:86	0.917
Follow up (mouths)	39.3 ± 11.2	40.2 ± 13.5	0.615

BMI, body mass index; OWHTO, open wedge high tibial osteotomy; UKA, unicompartmental knee arthroplasty.

### *Surgical Key Points*

**Fig. 1:**
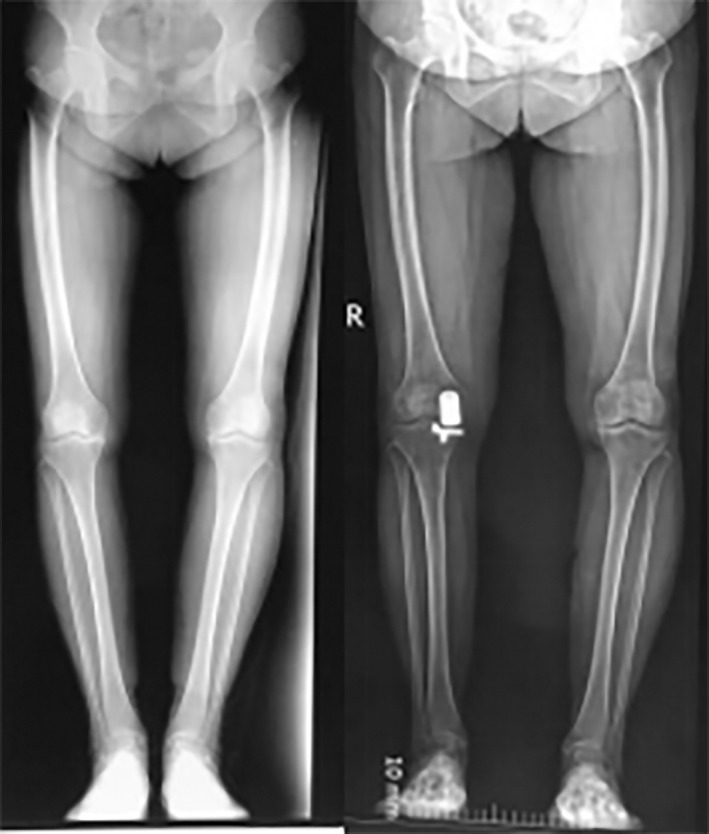
Typical case of unicompartmental knee arthroplasty operation.

For UKA, knee varus should be remediable in the physical examination, which means no medial collateral ligament (MCL) contracture was found. Patients were given general anesthesia. The medial parapatellar approach was undertaken. Pathological synovium, fat pads, medial meniscus, and osteophytes in the medial unicompartment were removed. After the osteotomy was done, bone cement was used to fix the prosthesis. After moving the knee repeatedly to make sure the joint function was reconstructed, suturing was carried out. It should be noted that surgical instruments for UKA and total knee arthroplasty (TKA) were both prepared before the operation. After exposure, the anterior cruciate ligament (ACL) and lateral unicompartmental knee cartilage (LUKC) were first examined. The UKA was replaced by TKA if any insufficiency or defect was found in the ACL or LUKC (Fig. 1).

**Fig. 2:**
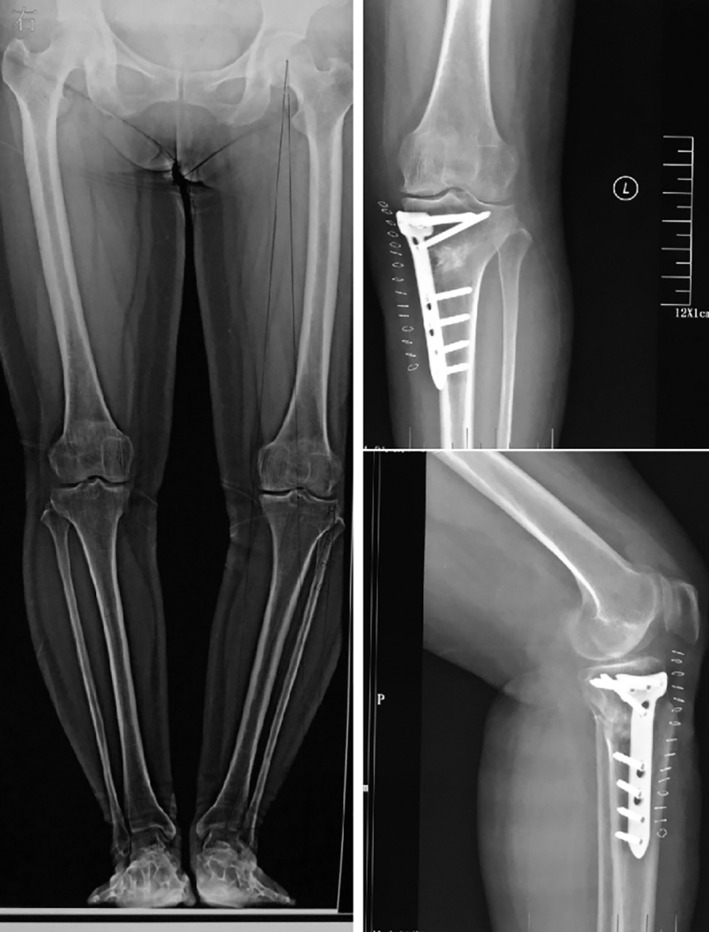
Typical case of open wedge high tibial osteotomy operation.

For OWHTO, X‐rays of the full length of the lower limbs were necessary. The tibial opening angle was calculated as α + 3°. α represents the acute angle made up by the tibial axis and the femur axis. Patients were given general anesthesia. The anteromedial approach to the upper tibial was adopted. After exposing the upper tibial, a Kirschner wire was used to determine the osteotomy line. A pendulum saw was used for the osteotomy and a spreader was used to spread the opening angle. Once the default opening angle was reached, the TomoFix internal fixation system was used to fix the tibial. If the opening angle was larger than 10°, an autogenous or artificial bone graft was carried out to prevent bone defects or ununion, while cases with opening angles smaller than 10° did not have any bone graft (Fig. 2).

### *Perioperative Management*

A buprenorphine transdermal patch was attached beneath the left clavical 1 day before the operation. Cefotiam 2 g was used 30 min before the operation by intravenous injection. Multipoint cocktail injection was performed in the operative area to relive postoperative local acute pain after prosthesis implantation or bone graft. The cocktail was made up of ketorolac tromethamine 30mg + dexamethasone 5 mg + bupivacaine 150 mg + adrenaline 0.25 mg, which was mixed up completely and then attenuated to 50 mL by normal saline. Patients were directed to do ankle pumps and quadriceps femoris contraction exercises after reviving from anesthesia. The drainage tube was open after being closed for 4 h, and was removed when the drainage volume was less than 50 mL in 24 h. Rivaroxaban 5 mg, qd, was taken in the postoperative 24 h to prevent DVT.

### *Outcomes Measurements*

#### *General Data*

General data of the patients was analyzed, including such perioperative data as operative time, incision length, dominant blood loss, and postoperative in‐bed time. The dominant blood loss represents the intraoperative dominant blood loss and postoperative drainage volume. The perioperative data was used to evaluate the outcome with enhanced recovery after surgery (ERAS).

#### *Hospital for Special Surgery Knee Scores*

The Hospital for Special Surgery (HSS) is a scoring system that is often used to evaluate knee function in the adult population. The HSS score system includes 6 aspects: pain, function, range of motion, myodynamia, absence of deformity, and stability. The score has a maximum of 100 points. A total score <59 is considered a poor score, 60–69 is fair, 70–84 is good, and 85–100 is excellent. In the present study, the HSS score was used to evaluate the postoperative recovery of knee function.

#### *Visual Analogue Pain Score*

The level‐walking visual analogue pain score (VAS) without weight‐bearing was determined at every follow up to evaluate the knee function. VAS is a pain score system with a range from from 0 to 10 that is based on the subjective feeling of the patients: 0 is analgesia and 10 is baryodynia; 1–3 is mild pain; 4–6 is moderate pain; and 7–10 is severe pain.

#### *Recovery for Low‐impact Sports*

A special questionnaire was designed to survey patients in regard to the ability to participate in low‐impact sports 1 year after the operation. In the questionnaire, five kinds of low‐impact sports, including cycling, exercise walking, jogging, dancing, and mountain climbing, were surveyed. If an individual's ability to participate in low‐impact sports could reach or surpass that before the operation, it was determined as the ideal recovery of this ability, and the patient received 1 point; otherwise, the patient received 0 points. The highest score that a patient can obtain from this questionnaire is 5, while the lowest is 0. A higher score indicates better recovery of the ability to participate in low‐impact sports. A total score ≤2 is defined as a poor score, 3 is fair, 4 is good, and 5 is excellent.

#### *Revision Case Analysis*

During the follow‐up period, any revisions caused for any reason were defined as revision cases. The revision cases were analyzed and reported in the present study.

### *Statistical Analysis*

Statistical analysis was performed with SPSS statistics software version 22.0 (IBM, Armonk, NY, USA). The numerical results are shown as mean ± standard deviation. A two‐sided paired Student *t*‐test was used to analyze the quantity results. The χ^2^‐test was carried out to analyze the quality of the results. The significant level was defined as ɑ = 0.05.

## Results

### *Follow‐up*

All patients were followed up at 1, 3, 6, and 12 months for the first year after the operation, and every 6 months from the second year. All patients were followed up by questionnaire survey, which included HSS score, VAS score, and low‐impact sports recovery. The average follow‐up periods of UKA and OWHTO groups were 39.3 ± 11.2 and 40.2 ± 13.5 months, and no difference was found between the two groups.

### *General Results*

General results are shown in Table 2. The operative time, incision length, dominant blood loss, and postoperative in‐bed time of the UKA group and the OWHTO group were 54.26 ± 10.14 min, 9.78 ± 1.53 cm, 90.52 ± 12.30 mL, and 3.49 ± 1.26 days and 67.52 ± 9.47 min, 10.95 ± 1.72 cm, 139.34 ± 17.76 mL, and 3.68 ± 1.03 days, respectively. The operative time, incision length, and dominant blood loss of the UKA group were less than those of the OWHTO group by 19.6%, 10.7%, and 35.1%, respectively, and the differences were significant (*P* < 0.05). However, no significant difference was found in postoperative in‐bed time between the two groups (*P* ≥ 0.05).

**TABLE 2:** Comparison on the general data between the two groups

	UKA group (*n* = 83)	OWHTO group (*n* = 109)	*P‐*value
Operative time (min)	54.26 ± 10.14	67.52 ± 9.47	0.000*
Incision length (cm)	9.78 ± 1.53	10.95 ± 1.72	0.000*
Dominant blood loss (mL)	90.52 ± 12.30	139.34 ± 17.76	0.000*
Postoperative in‐bed time (day)	3.49 ± 1.26	3.68 ± 1.03	0.265

OWHTO, open wedge high tibial osteotomy; UKA, unicompartmental knee arthroplasty; *, the difference is significant.

### *Functional Evaluation*

The functional evaluation includes HSS score, level‐walking VAS score, and low‐impact sports recovery.

The HSS scores of the two groups are shown in Table 3. The preoperative HSS scores of the UKA group and the OWHTO group were 50.8 ± 6.2 and 52.3 ± 5.8, and no significant difference was found between the two groups. The HSS scores at 1 and 3 months postoperation for the UKA group were 76.3 ± 5.4 and 81.6 ± 6.9, and 72.6 ± 7.5, 78.5 ± 8.6 for the OWHTO group, respectively. The two parameters of the UKA group were higher than those of the OWHTO group, by 5.1% and 3.9%, respectively, and the differences were significant (*P* < 0.05). However, the HSS scores for the UKA group and the OWHTO group were 87.1 ± 7.4 and 84.8 ± 9.7 at 6 months, 89.5 ± 6.3 and 88.2 ± 8.8 at 12 months, and 91.7 ± 7.2 and 90.6 ± 8.7 at 24 months postoperation and no differences were found in the HSS scores between the two groups from 6 months postoperation (*P* ≥ 0.05).

**TABLE 3:** Comparison on the HSS scores between the two groups

	UKA group (*n* = 83)	OWHTO group (*n* = 109)	*P‐*value
Preoperative	50.8 ± 6.2	52.3 ± 5.8	0.0865
1 month postoperative	76.3 ± 5.4	72.6 ± 7.5	0.0001*
3 months postoperative	81.6 ± 6.9	78.5 ± 8.6	0.0062*
6 months postoperative	87.1 ± 7.4	84.8 ± 9.7	0.0639
12 months postoperative	89.5 ± 6.3	88.2 ± 8.8	0.2346
24 months postoperative	91.7 ± 7.2	90.6 ± 8.7	0.3394

OWHTO, open wedge high tibial osteotomy; UKA, unicompartmental knee arthroplasty; *, the difference is significant.

The level‐walking VAS scores of the two groups are shown in Table 4. The preoperative VAS scores of the UKA group and the OWHTO group were 5.8 ± 1.6 and 5.6 ± 2.2, and no significant difference was found (*P* ≥ 0.05), while the 1‐month postoperative VAS scores of the UKA group and the OWHTO group were 4.3 ± 1.8 and 4.9 ± 2.1, respectively, of which the UKA group was lower than the OWHTO group by 12.2%, and the difference was significant (*P* < 0.05). The VAS scores at 3, 6, 12, and 24 months postoperation of the UKA group were 2.6 ± 1.2, 1.9 ± 1.2, 0.8 ± 0.7, and 0.5 ± 0.6, while those of OWHTO group were 2.9 ± 1.8, 2.2 ± 1.5, 1.0 ± 0.8, and 0.5 ± 0.7. No differences were found between the two groups from 3 months postoperation (*P* ≥ 0.05).

**TABLE 4:** Comparison on the level‐walking VAS scores between the two groups

	UKA group (*n* = 83)	OWHTO group (*n* = 109)	*P‐*value
Preoperative	5.8 ± 1.6	5.6 ± 2.2	0.4668
1 month postoperative	4.3 ± 1.8	4.9 ± 2.1	0.0385*
3 months postoperative	2.6 ± 1.2	2.9 ± 1.8	0.1684
6 months postoperative	1.9 ± 1.2	2.2 ± 1.5	0.1254
12 months postoperative	0.8 ± 0.7	1.0 ± 0.8	0.0719
24 months postoperative	0.5 ± 0.6	0.5 ± 0.7	1.0000

OWHTO, open wedge high tibial osteotomy; UKA, unicompartmental knee arthroplasty; *, the difference is significant.

The low‐impact sports recovery of the two groups is shown in Table 5 and Table 6. Only 14.5% (12:83) of patients in the UKA group and 15.6% (17:109) of patients in the OWHTO group could achieve excellent recovery in low‐impact sports. The good, fair, and poor results in the UKA group were 38.6% (32:83), 34.9% (29:83), and 12.0% (10:83), while those in the OWHTO group were 37.6% (41:109), 34.9% (38:109), and 11.9% (13:109). The average points of the UKA group and the OWHTO group were 3.46 ± 1.11 and 3.50 ± 1.07, respectively, and no significant difference was found (*P* ≥ 0.05). For the UKA group, 4.8% (4:83) of patients could not achieve ideal recovery in cycling, while 22.9% (19:83) could not in exercise walking, 20.5% (17:83) in jogging, 43.4% (36:83) in dancing, and 62.7% (52:83) in mountain climbing. For the OWHTO group, 5.50% (6:109) of patients could not achieve ideal recovery in cycling, while 11.9% (13:109) could not in exercise walking, 23.9% (36:109) in jogging, 33.9% (37:109) in dancing, and 75.2% (82:109) in mountain climbing.

**TABLE 5:** Comparison on the low‐impact sports recovery between the two groups

	UKA group (*n* = 83)	OWHTO group (*n* = 109)	*P‐*value
5 points	12	17	‐
4 points	32	41	‐
3 points	29	38	‐
≤2 points	10	13	‐
Average	3.46 ± 1.11	3.50 ± 1.07	0.8009

OWHTO, open wedge high tibial osteotomy; UKA, unicompartmental knee arthroplasty.

**TABLE 6:** One‐year postoperative abilities of low‐impact sports lower than those of preoperative ones of the two groups

	UKA group (*n* = 83)	OWHTO group (*n* = 109)
Cycling	4	6
Exercise walking	19	13
Jogging	17	26
Dancing	36	37
Mountain climbing	52	82

OWHTO, open wedge high tibial osteotomy; UKA, unicompartmental knee arthroplasty.

### *Complications and Revision Case Analysis*

For the OWHTO group, no infection, bone fracture, bone nonunion, fixation failure, DVT, or revision occurred during the hospitalization and follow‐up period. For the UKA group, no prosthetic loosening, periprosthetic fracture, or DVT occurred during the hospitalization and follow‐up period, while there was one case of infection (1.2%) and one case of subluxation of the liner (1.2%), both of which resulted in revision. The revision case analysis follows.

**Fig. 3:**
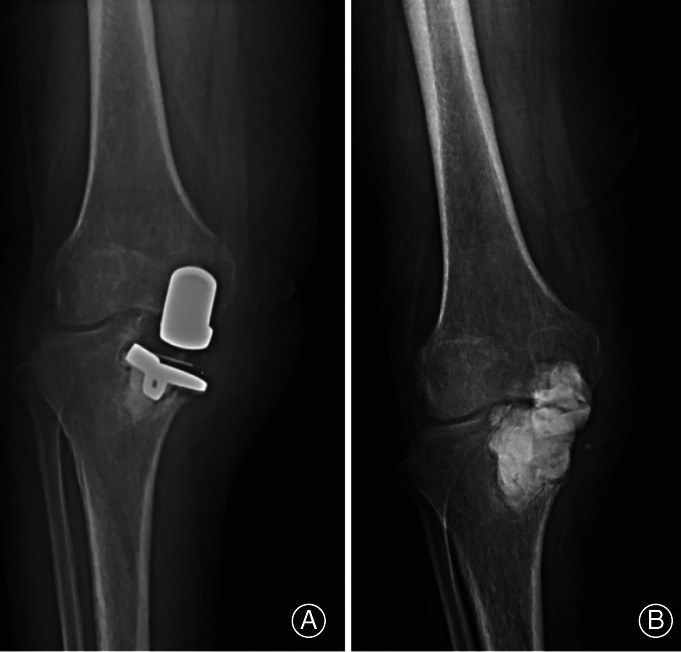
Revision case caused by infection: (A) prosthesis infection after unicompartmental knee arthroplasty; and (B) treated by cement spacing operation.

Revision Case 1, female, 58 years, caused by infection: The operative time was 56 min, the incision length was 9.6 cm, the dominant blood loss was 92 mL, and the postoperative time was 4 days. No wound complications were found during the first postoperative week, while some hemorrhagic exudation could be found on the surface when wound dressing. The patient treatment involved direction to rest in bed, enhancing wound care, and cefotiam *via* intravenous injection. The exudation disappeared 4 days later. The stitches were removed 1 week after the operation, and no anomaly, such as infection, exudation or malunion, was found. However, the patient came to our department again 2 months after discharge with yellow, thick exudation from the inferior margin of the wound. X‐ray showed the prosthesis loosening and tibial plateau collapse, and the bacterial culture revealed *Staphylococcus aureus*. The diagnosis was prosthetic loosening, tibial plateau collapse, and periprosthetic infection (PJI), all of which were the parameters for revision. The patient was treated with antibiotics and a cement spacing operation, and was prepared for two‐stage revision by TKA (Fig. 3).

**Fig. 4:**
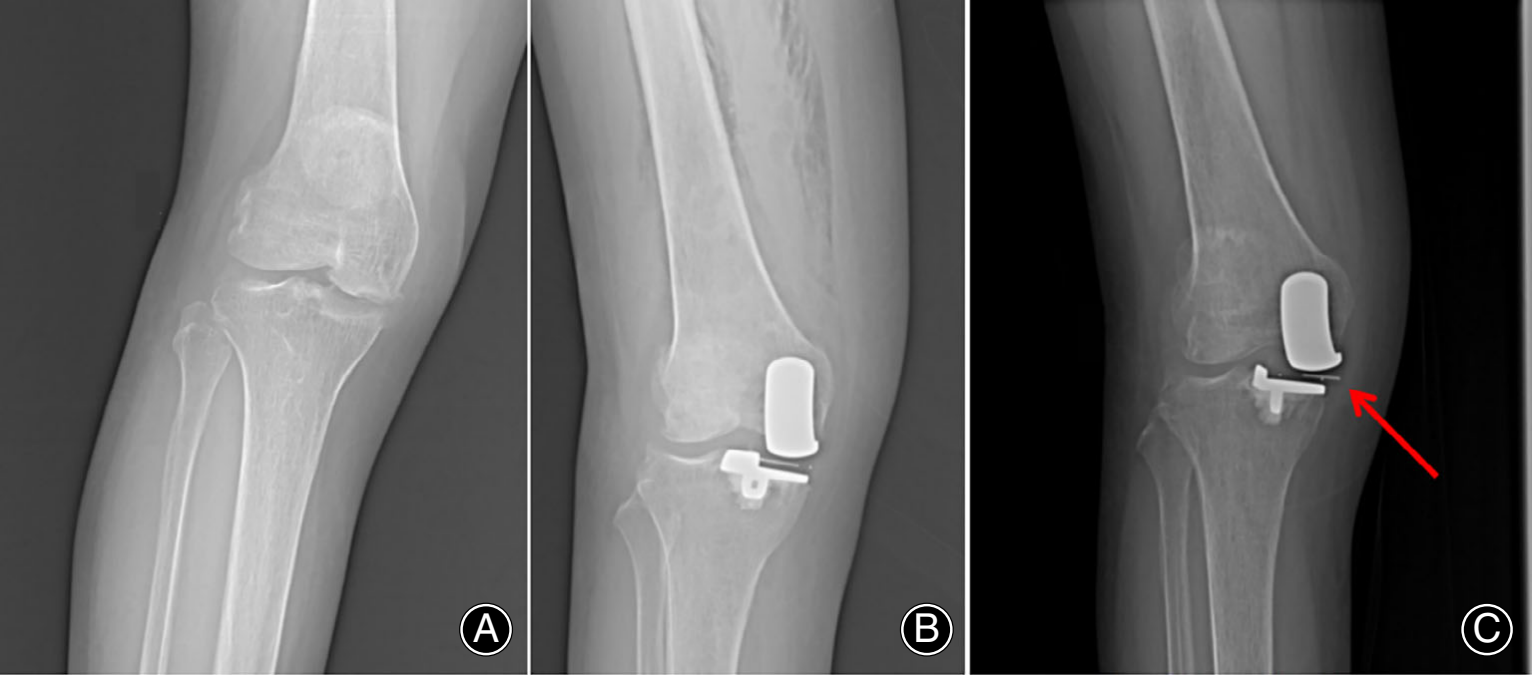
The revision case caused by liner subluxation: (A) preoperative X‐ray; (B) postoperative X‐ray; and (C) liner subluxation and collision with the medial collateral ligament.

Revision Case 2, female, 52 years, mobile‐bearing prosthesis, caused by subluxation. The operative time was 58 min, the incision length was 8.6 cm, the dominant blood loss was 83 mL, and the postoperative time was 3 days. No complications were found during the hospitalization period. The patient returned to hospital 7 months after discharge, complaining of interior knee pain. MCL loosening could be found during physical examination. The patient said that the symptoms appeared during dancing. in which repeated knee rotations were needed. X‐ray showed that the medial knee space was decreased, with liner subluxation, and collision between the liner and the MCL. The diagnosis were MCL loosening and liner subluxation. MCL reconstruction and liner reduction were originally planned. However, during the operation, overwear was found on the liner, which was the main parameter for liner replacement (Fig. 4).

## Discussion

The present study compared the effects of UKA and OWHTO in treating MKOA in patients under 60 years. The results showed that the operative time, incision length, and dominant blood loss of the UKA group were all superior to those of the OWHTO group, and the differences were significant (*P* < 0.05). For the HSS score and VAS score, the UKA group was superior to the OWHTO group in the early stages postoperation, and the differences were significant (*P* < 0.05), while no difference was found in the middle to long‐term stages of postoperation (*P* ≥ 0.05), indicating that UKA may be superior to OWHTO in ERAS. This result is similar to those of a previous report^18^. However, in the present study, no revision case occurred in the OWHTO group, while two revisions occurred in that UKA group (2.4%), indicating that OWHTO may result in better survivorship.

In regard to the recovery to participate in low‐impact sports, at 1 year after the operations, only 14.5% (12:83) of patients in the UKA group and 15.6% (17:109) of patients in the OWHTO group could achieve ideal recovery in all five kinds of sports, indicating that most patients undergoing UKA or OWHTO would lose some ability to participate in low‐impact sports 1 year after the operation. Mountain climbing was the sport in which most patients could not achieve ideal recovery in both groups. We believe the reason is that among the five sports, mountain climbing is the one that demands good function in terms of both stability and strength of the knee, which requires greater recover time.

The first indications for UKA were provided by Scott in 1989, including: (i) patients should not be obese or extremely active; (ii) less than 15° of angular knee deformity that is passively correctable to neutral; (iii) disabling pain should be absent at rest; (iv) preoperative 90° flexion arc should be demonstrated with less than 5° of flexion contracture; and (v) both crutiate ligaments should be intact. He also pointed out that the best results can be expected in elderly patients, but younger patients can also benefit from UKA^19^. However, Goodfellow, who was one of the designers of the Oxford UKA prosthesis, presented a new version of indications for UKA based on Scott's indications in 2006; this is the most‐widely used now and includes: (i) bone‐on‐bone anteromedial osteoarthritic wear pattern; (ii) ligamentously normal knee with an intact ACL; (iii) correctable varus deformity; and (iv) well‐maintained, normal lateral joint space on valgus stress view radiograph^20^. Goodfellow also raised the contraindications for UKA, including: (i) inflammatory arthropathy; (ii) previous high tibial osteotomy (opening or closing wedge); (iii) ACL deficiency; (iv) MCL contracture with inability to correct the varus deformity; (v) weight‐bearing cartilage wear of the lateral compartment; and (vi) severe patellofemoral arthrosis with lateral facet disease, lateral subluxatiion, and trochlear grooving. Compared with the Scott version, the indications for UKA in the Goodfellow version mainly changed the following three points: (i) the limitations on obese and activity of the patients were removed; (ii) the importance of the ACL was emphasized, while the PCL was not under consideration anymore; and (iii) demand on the completeness of the lateral compartment was added.

Because the indications of UKA and OWHTO are similar^21^, the two operations were frequently compared in regard to the effects in treating MKOA. In earlier studies, UKA showed significant superiority to HTO in both outcomes and survivorship^22^, ^23^. However, due to the improvements in the internal fixation system and the surgical technique of OWHTO, and the extension of the indications of UKA, the clinical outcomes of OWHTO are now comparable to those of UKA^24^, ^25^. In fact, there has been some research pointing out the controversy of the current indications of UKA. Kandil's report showed that obese (BMI > 30) and morbidly obese (BMI > 35) patients have a significantly higher risk in relation to both complication and revision rates^26^. Takeuchi *et al*. believe that OWHTO is more suitable for active people than UKA^27^, despite Crawford *et al*. reporting that activity level does not affect survivorship of UKA^15^. Sever *et al*. even state that “we do not recommend the use of Oxford UKA surgery commonly in the treatment of medial compartment osteoarthritis” (page 239) and Schroer *et al*. that “The unacceptable rate of failure with the Oxford knee implant has led the principal investigator to discontinue its use in practice” (page 3538)^16^, ^17^. In the present study, we also found that the revision rate of the UKA group was higher than that of the OWHTO group, even though the two groups had similar outcomes and function recovery, and UKA showed a potential superiority in ERAS.

However, we do not consider that UKA should be denied. We believe the reason for the current controversy regarding UKA is that different surgeons have different understandings of the indications of UKA, and the non‐uniform indications lead to various results. In fact, we believe that UKA has a different requirement to OWHTO in regard to the stability of the knee. Both UKA and OWHTO demand good preoperative stability of the knee, including an intact ACL and correctable varus deformity. As shown in Revision Case 2, postoperative instability caused by MCL insufficiency led to liner subluxation and subsequent revision, which would not occur in OWHTO.

Similar to the Revision Case 2 in the present study, Kawaguchi *et al*. also reported two cases of liner dislocation of Oxford mobile‐bearing prostheses while patients were rolling over in their sleep^28^. They believed that MCL instability and the valgus position of the knee were the reasons, and suggested that the valgus and mild knee flexion should be reproduced in operations, so that the liner could be dislocated into the intercondylar ridge. However, we do not support this solution. One of the advantages of UKA is that it barely changes the force line, length, and gait of lower limbs, which results in better proprioception for patients than OWHTO^29^, ^30^. If valgus reproduction is necessary during UKA, why do we not just choose OWHTO instead?

Thus, based on the present study and previous research, we suggest two modifications to the Goodfellow version of indications and cotraindications of UKA. First, demand on activity of patients should not be removed and extremely active patients have a higher risk of damage, which may cause instability of knee, and lead to revision. Second, not only MCL contracture but also MCL insufficiency should be added to the contraindications of UKA, especially for the mobile‐bearing UKA.

In general, in this study, no significant differences were found in middle‐term to long‐term outcomes between the UKA group and the OWHTO group. However, UKA demonstrated faster functional reconstruction but a potential higher revision rate. We believe that besides age, the preoperative and postoprative stability, which is determined by the activity of patients, should also be considered carefully before a UKA operation is carried out. The varying results reported by previous studies on the outcomes of UKA may be caused by the non‐uniform indications by different surgeons. The current indications for UKA should be modified.

A limitation of the present study is that the cases are from two hospitals, which means that the operations were carried out by different surgeons. However, to reduce the effect as far as possible, the operations in the study were all carried out by chief surgeons. In future research, we will collect more cases and cases with longer follow up carried out by one of the chief surgeons to prove the results.

In conslusion, no significant differences were found in mid‐term to long‐term outcomes between UKA and OWHTO. However, besides age, preoperative and postoperative stability are also determining factors in operation choice, and should be added to indications of UKA. For patients who are not active and have less risk in terms of postoperative instability, UKA could be better for the superiority in ERAS, while for those who are more active, OWHTO would be better.
